# Comparison of the safety and efficacy of three superficial skin closure methods for multi-layer wound closure in total knee arthroplasty: a multicenter, prospective, randomized controlled trial

**DOI:** 10.1186/s42836-024-00271-1

**Published:** 2024-09-11

**Authors:** Te Liu, Ye Tao, Runkai Zhao, Yanfan Hua, Zeyu Feng, Qingyuan Zheng, Guoqiang Zhang, Lei Geng, Jun Fu, Wenwei Qian, Ming Ni, Weijun Wang

**Affiliations:** 1 Department of Orthopedics, The First Medical Center, Chinese PLA General Hospital, Beijing, 100853 China; 2 Department of Orthopedics, The Fourth Medical Center, Chinese PLA General Hospital, Beijing, 100036 China; 3 Medical School of Chinese PLA, Beijing, 100853 China; 4 Department of Orthopedic Surgery, The Affiliated Drum Tower Hospital of Nanjing University of Chinese Medicine, Nanjing, 210008 China; 5 Department of Orthopedic Surgery, Peking Union Medical College Hospital, Peking Union Medical College, Chinese Academy of Medical Science, PekingBeijing, 100730 China

**Keywords:** Total knee arthroplasty, Infection, Wound closure, Skin adhesive, Subcuticular suture

## Abstract

**Background:**

Good wound healing is critical to infection prophylaxis and satisfactory rehabilitation in Total Knee Arthroplasty (TKA). Currently, two techniques, i.e., barbed continuous subcuticular suture without skin adhesive or combined use skin adhesive (n-butyl-2) are being used for superficial wound closure of TKA. While a new skin adhesive (2-octyl) with self-adhesive mesh has been employed as an alternative to conventional surgical skin closure in TKA, its superiority, especially in reducing wound complications and improving wound cosmetic outcomes has not been investigated. This study aimed to compare 2-octyl, n-butyl-2, and no skin adhesive in terms of safety and efficacy in TKA superficial wound closure.

**Methods:**

We conducted a multicenter, prospective, randomized controlled study in 105 patients undergoing primary TKA between May 2022 and October 2023. Each patient’s knee was randomized to receive 2-octyl, n-butyl-2, or no skin adhesive skin closure with all using barbed continuous sutures in deep tissue. Wounds were followed 1, 3, 5 days, 2, 6 weeks, and 3 months after surgery. Wound discharge, complications, cosmetic outcomes, patient satisfaction, and wound-related costs were compared among these three methods.

**Results:**

Wound discharge was less in 2-octyl group and n-butyl-2 group than in non-adhesive group at 1 day, with the discharge only being less in 2-octyl group than in the non-adhesive group at day 3 and day 5 days (*P* < 0.05). There was no statistical difference in the incidence of other wound complications among the groups (*P* > 0.05). The 2-octyl group achieved better cosmetic effects than the other two groups in 6 weeks and 3 months (*P* < 0.05). Compared to the non-adhesive group, 2-octyl group scored higher in overall patient satisfaction score in 2 weeks and incurred lower costs (*P* < 0.05).

**Conclusions:**

Skin closure in TKA using 2-octyl adhesive material showed superiority when compared to no skin adhesive or n-butyl-2, in reducing wound discharge, improving the cosmetic outcomes, without increasing wound complications. In addition, the use of 2-octyl yielded better patient satisfaction and also was less costly compared to no skin adhesive. Our study exhibited that 2-octyl was a safe and effective wound closure technique for patients undergoing TKA.

**Trial registration:**

This study has been registered at Clinical Trials. Gov (No. ChiCTR210046442).

## Background

The number of total knee arthroplasty (TKA) is projected to be on the rise in the coming years [1–3]. Effective wound closure can promote the healing of wounds after TKA, reduce wound complications such as wound discharge, and improve wound cosmetic outcomes [1–8]. Orthopedists have been trying to use new materials and methods for wound closure to achieve these goals [3, 7]. Although barbed continuous suture for TKA deep tissue closure has good track records, no consensus has been reached regarding the best method for superficial skin suture [1, 9].

In recent years, skin adhesives for superficial wound closure have been introduced as a substitute for or supplement to traditional closure techniques [9, 10], such as n-butyl-2 and the new skin adhesive 2-octyl [11, 12]. The former is n-butyl-2-cyanoacrylate and has been widely used in clinical practice for more than 50 years. This adhesive is relatively hard, and reportedly could not effectively reduce the incidence of wound drainage [13, 14]. 2-Octyl is a breathable, self-adhesive mesh covering wounds and consists of a flexible self-adhesive polyester mesh and a 2-octyl cyanoacrylate skin adhesive, which, through the mesh, distributes tension uniformly at the edge of the wound. Theoretically, it provides a mechanical barrier to bacteria and also renders the wound water-tight, thereby minimizing wound discharge, and lowering the risk of infection early after surgery [11, 15–19]. 2-octyl has yielded favorable clinical results in surgical wound closure in a multitude of fields, such as the closure of full-thickness surgical wounds [20], abdominoplasty [16], reduction mammaplasty [12], spinal fusion surgery [21], metastatic osteopathy [22], among others. In contrast to the incisions, the post-TKA wound is subject to high mechanical tension during rehabilitation. Whether 2-octyl with mesh reduces wound discharge or drainage to promote wound healing has not been well investigated. In a randomized controlled trial on TKA, Keun et al. concluded that 2-octyl was a useful substitute for subcuticular suture, and patients receiving 2-octyl had better wound margin coaptation but the cosmetic outcomes were similar to those of subcuticular suture [11]. On the other hand, Kavin et al. showed that 2-octyl attained better cosmetic outcomes in TKA [19]. A systematic review pointed out that it is impossible to draw a clear conclusion as to whether the use of skin adhesives can accomplish good cosmetic outcomes [14]. In addition, a multicenter study will help confirm the utility of 2-octyl in skin closure in patients undergoing TKA.

This study aimed to conduct a multicenter, prospective, randomized controlled trial to study the safety and effectiveness of using 2-octyl in TKA wound superficial closure, and compared it with n-butyl-2 and no skin adhesive, in terms of (1) wound discharge and other wound complications, (2) wound cosmetic outcomes (POSAS, HWES, and VSS scores) and (3) patient’s overall satisfaction with wound closure and incision-related cost etc. Our hypothesis was that 2-octyl was superior to n-butyl-2 or no skin adhesive, especially in reducing postoperative wound discharge in TKA wound closure.

## Methods

### Study design

This study was approved by the Ethics Committee of PLA General Hospital. We conducted a multicenter, prospective, randomized controlled study in the First Medical Center of the PLA General Hospital, the Fourth Medical Center of the PLA General Hospital, and the Nanjing Drum Tower Hospital, the affiliated hospital of Nanjing University Medical School.

### Inclusion and exclusion criteria

Inclusion criteria included: (1) patients who had undergone primary unilateral TKA; (2) aged 18 to 75 years; (3) no trauma or infection present at the surgical site; (4) physically and mentally healthy, able to participate in and cooperate with the trial. Exclusion criteria were: (1) having received multiple operations at the surgical site; (2) having been found to be allergic to wound closure materials before the trial; (3) patients who were prone to scar formation (Those who had a personal history of having developed keloid or hypertrophic scar formation.); (4) patients whose blood pressure or glucose were poorly controlled; (5) severe malnutrition; (6) rheumatoid diseases, connective tissue diseases and other immunodeficiency diseases; (7) hemophilia, vascular diseases of affected limbs; (8) body mass index (BMI) > 35; (9) patients on corticosteroids, anticoagulants, immunosuppressants and other drugs; (10) those who refused to participate in the trial.

### Randomization

In a double-blind, simple randomization procedure, eligible patients were identified the night before surgery by an investigator. The random numbers were generated by a surgeon participating in the experiment via SPSS27.0 software and patients were divided into three groups based on the numbers. And then put a note with grouping information into a sealed opaque envelope numbered according to random numbers in advance. Patients from whom the informed consent was obtained were randomly assigned, at 1:1:1, into one of the three groups: i.e., 2-octyl, n-butyl-2, and no skin adhesive groups. The surgeon was then notified of the assignment to prepare materials for surgical wound closure. To achieve double blindness, grouping, and wound closure were performed by doctors who were not involved in subsequent wound evaluation, and all photos of applied dressings were collected and numbered without grouping information during the evaluation of wound discharge. During follow-up, the other two doctors and the patients who evaluated the cosmetic results of the wound were not informed of the name and group information of each patient.

**Fig. 1:**
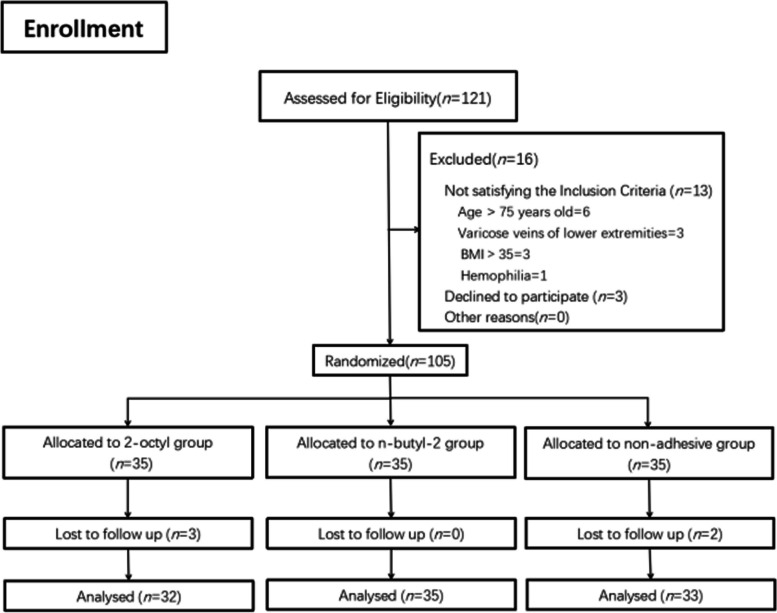
Random grouping flow chart (121 patients were evaluated for eligibility, 105 patients were included and grouped on random basis, and 100 patients entered the final analysis)

From May 2022 to October 2023, 121 patients underwent primary TKA for knee osteoarthritis at the aforementioned three hospitals, 16 patients were excluded and five patients were lost to the follow-up due to personal reasons. 100 patients entered the final analysis, each patient group containing patients from three different hospitals. The number of patients in one hospital was no more than 50% of the total number of patients in each group. (Fig. 1. outlines the patient flow of the study).

### Surgical technique

Preoperatively, antibiotics were routinely used to prevent infection. All patients were placed in the supine position and operated on by a senior surgeon using a standard midline anterior knee incision via medial parapatellar approach, with a tourniquet used during the operation. Tranexamic acid was given intravenously before skin incision and wound closure. All patients were administered antibiotics within 24 h after surgery and received standard pain management, anticoagulation, and rehabilitation.

### Wound closure

**Fig. 2:**
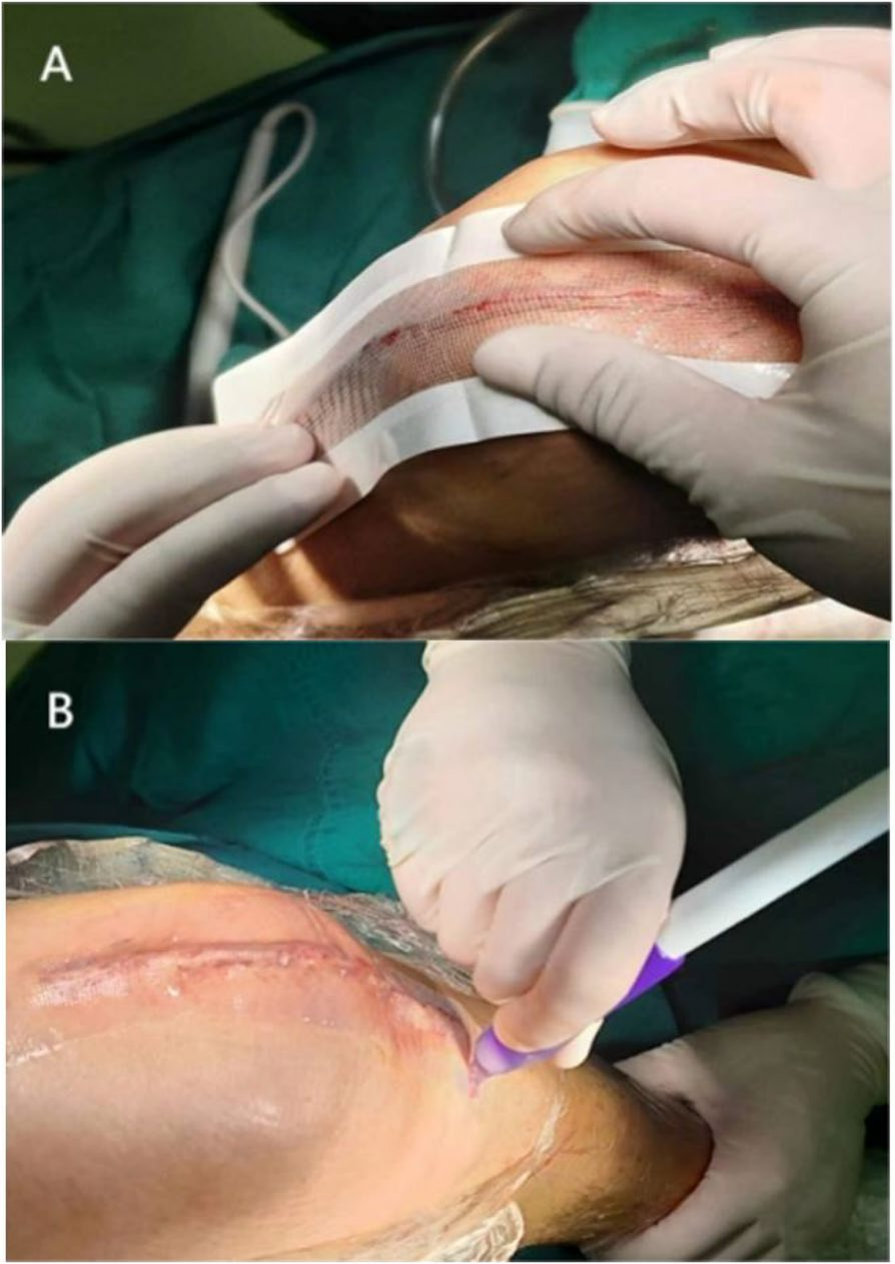
**A** The self-adhesive mesh of sufficient length was used to cover the edge of the skin wound with a margin of more than 1 cm. Gentle pressure was applied, with fingers, to make the mesh attach to skin snugly. **B** The 2-octyl skin adhesive was applied onto the entire length of the belt in a smooth and uniform Mesh

All wounds were sutured in three layers with standard continuous suture separately to reduce skin tension and align the edge of the wound. Capsule and subcutaneous tissues were closed with symmetric continuous suture separately by using absorbable barbed sutures of size 1 and size 0, respectively (STRATAFIX Symmetric PDS Plus, Ethicon LLC). After closure of the joint capsule, the joint was moved within a wide range to check whether the incision was exudating or not. If necessary, local sutures, such as polyester non-absorbable suture W4843 (Ethicon, LLC) or absorbable suture (type, 4/0; coated Vicryl Plus antibacterial suture Ethicon, LLC) was used for intermittent suture reinforcement to achieve good sealing of the incision suture. For closure of skin layers, two surgeons, using absorbable cosmetic sutures simultaneously (Knotless tissue control device STRATAFIX™ Sprial PGA-PCL Ethicon, LLC), performed continuous intradermal suture, from the middle point of the wound to both proximal and distal ends. When the end of the wound was reached, the thread was passed out of the skin beside the wound and placed 1–2 stitches backward. Afterward, the remaining suture was cut off without knotting. For the knee in the 2-octyl group (DERMABOND® PRINEO® 2-octyl cyanoacrylate skin adhesive (3.8 mL) with flexible self-adhesive polyether mesh (22 cm), Ethicon, Inc.) group, a mesh long enough to fully cover skin wound with a 1 cm allowance was used and liquid adhesive was applied to the mesh and was allowed to polymerize and dry (Care should be taken to make the cutting edges fit snugly so that the mesh surface adhered tightly to the skin without gaps) (Fig. 2). In the n-butyl-2 group, n-butyl-2 (n-butyl-2-Cyanoacrylate B. Braun Corp, Melsungen, Germany) was applied, thinly and evenly (about 5 mm wide), to the wound surface according to the manufacturer's instructions, and was left to set. In the non-adhesive group, the wound was finally wiped with alcohol gauze, and in the two adhesive groups, the wound was also cleaned with alcohol gauze before the application of skin adhesive. In all groups, standard disposable self-adhesive wound sterile dressings were applied after wound closure to evaluate postoperative wound discharge. All wound closures were done by attending physicians who are experienced in stitching the above-mentioned sutures and had been trained in the use of skin adhesive to avoid skill- or ability-related result deviations.

### Follow-up and data collection

Basic information about each patient was collected, including age, sex, body mass index (BMI), diabetes, hypertension, and CCI (Charlson Comorbidity Index) [23]. The laboratory findings (hemoglobin, albumin, platelets, C-reactive protein, erythrocyte sedimentation rate) before and on the first and third days after operation. The wound length (measured with a soft tape rule in mm at knee flexion of about 45° after suture completion). Postoperative hospital LOS was recorded. When the postoperative hospital stay was calculated in days, and time less than one day was counted as one day.

The wound was evaluated at 1 day, 3 days, 5 days, 2 weeks, 6 weeks, and 3 months postoperatively. If the wound dressing oozed, loosened, fell off or the patient felt uncomfortable, the wound dressing was changed immediately. In the skin adhesive group, the dressing was changed without sterilization, while in the non-adhesive group, conventional surgical dressing changes were required. If the surgical wound healed completely without discharge, the dressing was removed and the wound was directly exposed to the air 2 weeks after the operation, otherwise, the dressing change continued. The dressing was collected on the 1st, 3rd, and 5th day after operation for evaluation of wound discharge.

The primary outcome measure was the development of wound complications, including wound discharge and other wound complications.

In this study, wound discharge was defined as the presence of discharge strike-through onto the dressing. Prolonged wound discharge was a key risk factor for infection according to previous literature [18]. We defined wound discharge > 2 × 2 cm^2^ 72 h after surgery as Persistent Wound Drainage (PWD, Persistent Wound Drainage) [24, 25], and took photos of the discharge using standard photography techniques, then scanned and analyzed the pictures by employing open-source analysis software ImageJ, to estimate the area of each discharge (cm^2^). Other wound complications, including blisters, shallow infection [26], deep infection [27], wound dehiscence, ACD (erythema, eczema, or edema associated with adhesive use in the sutured area or to which the skin adhesive network adheres, depending on clinical symptoms, history, and wound appearance) [28, 29].

### The secondary outcome measures

Hollander wound evaluating score (HWES) was used at 2 and 6 weeks postoperatively (a 0–6 point scale, with 0 point indicating the best) [30]. VSS (Vancouver Scar Scale) was employed for assessing the wound 6 weeks and 3 months after surgery (a 0–14 point scale, 0 point indicating the best) [31]. Wound were rated 2 weeks, 6 weeks, and 3 months postoperatively on the Patient-Observer Scar Assessment Score (POSAS). The scale consists of two independent scales, i.e., the patient scar assessment score (PSAS) and observer score assessment score (OSAS), with a total score range of 6–60 [32].

For cosmetic evaluation of wound, all wound scores were compared with normal skin at comparable anatomical locations by two high-volume surgeons who were not involved in the study (not members of the research team), or by the patient himself or herself. The final score was the average score of the scores assigned by the two surgeons.

Patients’ overall satisfaction toward wound closure was assessed on the Likert scale to evaluate (ranging from 1 ~ 5 points, with 5 points indicating the best, [33] (options included very satisfied, satisfied, neutral, dissatisfied, and very dissatisfied). Wound closure-related costs were the sum of the cost of skin adhesive, wound care costs during hospitalization, including costs of dressing change, dressing, and wound care costs after discharge, including registration fees, cost of dressing, transportation expenses, etc.).

### Statistical analysis

SPSS (version 27.0, IBM Corp. Armonk, NY, USA) was used for statistical analysis, with significance set at *P* < 0.05. Shapiro-Wilke test was utilized to determine the significance of differences between groups, ANOVA was employed for data with normal distribution, and the Kruskal–Wallis test was conducted for non-normally distributed data. Chi-square or Fisher’s exact test was applied to analyze the differences among count data. Bonferroni correction was used to assess any significant difference among groups and post-hoc analysis of chi-square was performed for multiple comparisons. The power calculation of this effectiveness study was based on the average discharge areas on the first postoperative day. Based on the previously published literature and pre-experiment results, the true difference between the means was assumed to be at least one standard deviation of the variable value, a sample size of 22 (per group) would provide a power of 0.9 to detect a significant difference of 5%. Therefore, three groups involving 90 patients (30 per group) would allow a 20% loss to follow-up [34].

## Results

The basic data of patients in each group are shown in Table 1. The laboratory results are shown in Table 2.

**Table 1:** Demographics and baseline statistics

**Characteristics**	**Group 1** **(2-octyl)**	**Group 2** **(n-butyl-2)**	**Group 3** **(non-adhesive)**	***P*-** **value**
**Age**^**a**^** (years)**	66.09 ± 6.20	66.80 ± 5.44	64.36 ± 8.09	0.540
**Gender**^**c**^**: male (%)**	8(25)	11(31)	8(24)	0.763
**Side**^**c**^**: right (%)**	17(53)	21(49)	20(61)	0.605
**BMI**^**a**^** (kg/m**^**2**^**)**	26.58 ± 4.04	27.15 ± 2.99	27.33 ± 3.21	0.659
**Charlson Comorbidity Index**^**b**^	1(1)	1(1)	1(1)	0.921
**Diabetes mellitus**^**c**^** (%)**	2(6)	4(11)	4(12)	0.767
**Hypertension disease**^**c**^** (%)**	12(38)	17(49)	19(58)	0.268
**Smoking (%)**	7(22)	4(11)	1(3)	0.060
**Length of wound**^**a**^** (cm)**	14.81 ± 1.54	15.45 ± 1.20	15.44 ± 1.27	0.079
**Tourniquet time**^**a**^** (min)**	66.00 ± 12.04	72.31 ± 16.60	74.18 ± 16.83	0.091

^a^Data are presented as means ± standard deviations

^b^Data are presented as median (IQR)

^c^Data are presented as numbers (percentage) of patients

**Table 2:** Comparison of three groups in terms of clinical parameters

**Parameters**	**Group 1** **(2-octyl)**	**Group 2** **(n-butyl-2)**	**Group 3** **(non-adhesive)**	***P*-value**
**Preoperative**				
**Hb (g/L)**	130.38 ± 13.74	129.43 ± 10.39	127.64 ± 12.27	0.669
**Alb (g/L)**	39.96 ± 2.77	40.25 ± 2.66	41.08 ± 2.46	0.207
**PLT (10**^**9**^**/L)**	216.75 ± 52.96	212.57 ± 64.94	207.06 ± 55.17	0.797
**CRP (mg/L)**	2.66 ± 3.26	5.76 ± 10.42	2.67 ± 3.69	0.253
**ESR (mm/H)**	11.38 ± 6.56	12.17 ± 13.37	10.64 ± 6.37	0.579
**Postoperative day 1**				
**Hb (g/L)**	118.66 ± 13.34	122.17 ± 12.93	119.85 ± 12.17	0.328
**Alb (g/L)**	36.81 ± 2.83	37.53 ± 2.86	38.69 ± 2.62	0.026^a^
**PLT (10**^**9**^**/L)**	199.66 ± 57.52	207.11 ± 60.76	201.39 ± 53.25	0.855
**CRP (mg/L)**	21.00 ± 18.20	22.95 ± 22.10	21.22 ± 14.95	0.792
**ESR (mm/H)**	23.69 ± 20.38	17.22 ± 15.20	18.61 ± 12.40	0.224
**Postoperative day 3**				
**Hb (g/L)**	112.74 ± 14.37	111.36 ± 23.28	115.13 ± 15.67	0.667
**Alb (g/L)**	37.05 ± 3.74	35.99 ± 7.17	37.69 ± 2.61	0.749
**PLT (10**^**9**^**/L)**	193.08 ± 62.56	196.25 ± 59.20	177.61 ± 48.85	0.483
**CRP (mg/L)**	27.63 ± 24.92	30.92 ± 34.52	31.83 ± 42.80	0.734
**ESR (mm/H)**	22.00 ± 14.03	19.76 ± 14.23	19.73 ± 10.61	0.768

*Hb* Hemoglobin, *Alb* Albumin, *CRP* C-reactive protein, *ESR* Erythrocyte sedimentation rate, *PLT* Platelets

^a^Indicates statistical significance. (statistically significant difference)

**Fig. 3:**
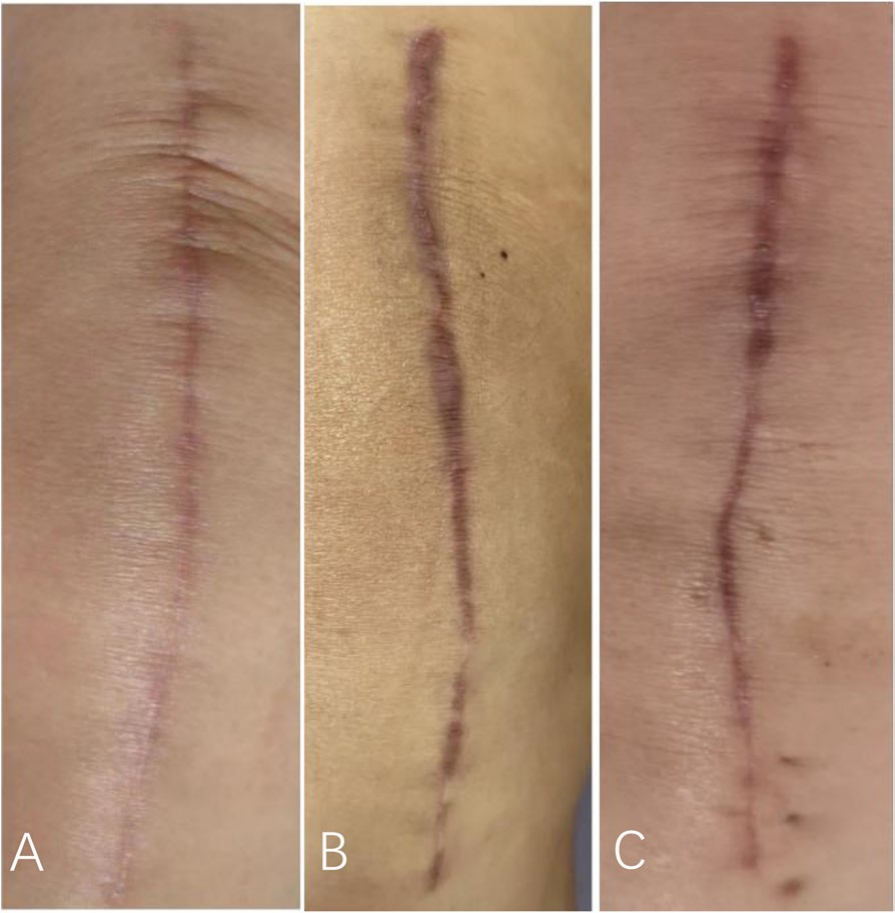
Incisions of 2-octyl group (**A**), n-butyl-2 group (**B**) and non-adhesive group (**C**) at 3 months

Multiple comparisons showed that the discharge area was smaller in 2-octyl group and n-butyl-2 group than in the non-adhesive group on postoperative day 1 after Bonferroni adjustment (*P* = 0.002; *P* = 0.000) (Table 3). The discharge area was significantly lower only in the 2-octyl group than in the non-adhesive group on the third day (*P* = 0.002) and fifth day (*P* = 0.003). 2-octyl group had the lowest incidence of PWD and multiple comparisons suggested that the incidence was significantly lower in 2-octyl group than in the non-adhesive group (*P* < 0.05). There was no statistically significant difference in the incidence of other wound complications among the groups (*P* > 0.05) (Table 3). The wound appearance at 3 months is shown in Fig. 3 and cosmetic outcomes are presented in Table 4. Bonferroni adjustment showed that the VSS score of 2-octyl group was significantly lower at 6 weeks than that of the other two groups (*P* = 0.025; *P* = 0.005) and the OSAS score of 2-octyl at 6 weeks was significantly lower than that of n-butyl-2 group (*P* = 0.050). The OSAS of the 2-octyl group at 3 months was significantly lower than that of the other two groups (*P* = 0.035; *P* = 0.020). The satisfaction score of the 2-octyl group at 2 weeks was significantly higher than that of the non-adhesive group (*P* = 0.028) (Table 5). The LOS of the 2-octyl group was significantly shorter than that of n-butyl-2 group (*P* = 0.012) and wound closure-related costs are listed in Table 5.

**Table 3:** Wound complications

**Characteristics**	**Group 1** **(2-octyl)**	**Group 2** **(n-butyl-2)**	**Group 3** **(non-adhesive)**	***P*-value**
**Discharge area day 1**^a^ **(cm**^**2**^**)**	1.69 ± 2.16	1.20 ± 2.29	11.96 ± 14.80	< 0.001^c^
**Discharge area day 3**^a^ **(cm**^**2**^**)**	0.55 ± 1.31	2.67 ± 9.97	8.80 ± 15.71	0.003^c^
**Discharge area day 5**^a^ **(cm**^**2**^**)**	0.16 ± 0.90	0.85 ± 2.10	3.54 ± 8.27	0.004^c^
**PWD**^b^	1(3)	5(14)	9(27)	0.018^c^
**Dehiscence**^b^	0(0)	1(3)	0(0)	1.000
**Superficial infection**^b^	0(0)	0(0)	0(0)	1.000
**Deep wound infection**^b^	0(0)	0(0)	1(3)	0.650
**ACD**^b^	0(0)	1(3)	0(0)	1.000
**Blister**^b^	0 (0)	0(0)	1(3)	0.650

*PWD* Persistent Wound Drainage, *ACD *Allergic contact dermatitis

^a^Data are presented as means ± standard deviations

^b^Data are presented as numbers (percentage) of patients

^c^Indicates statistical significance

**Table 4:** Comparisons of VSS, HWES and POSAS among the three groups

**Outcomes**	**Group 1** **(2-octyl)**	**Group 2** **(n-butyl-2)**	**Group 3** **(non-adhesive)**	***P*-value**
**2W**				
OSAS	13.06 ± 5.08	13.66 ± 2.49	14.15 ± 3.64	0.371
PSAS	19.06 ± 8.75	23.29 ± 12.55	20.06 ± 9.52	0.426
HWES	1.34 ± 1.41	1.57 ± 1.24	1.42 ± 1.50	0.663
**6W**				
VSS	5.50 ± 2.66	7.00 ± 2.40	7.42 ± 3.00	0.013^a^
OSAS	15.50 ± 3.50	17.43 ± 4.18	17.03 ± 4.50	0.030^a^
PSAS	19.06 ± 7.88	21.91 ± 8.66	23.15 ± 9.62	0.290
HWES	1.28 ± 0.96	1.91 ± 1.62	1.72 ± 1.61	0.415
**3 M**				
VSS	5.09 ± 2.58	5.57 ± 2.82	5.55 ± 2.59	0.677
OSAS	12.78 ± 3.42	17.40 ± 6.58	16.79 ± 5.86	0.010^a^
PSAS	17.87 ± 8.07	20.80 ± 9.57	21.44 ± 10.47	0.411

*VSS* Vancouver Scar Scale, *POSAS* Patient and Observer Scar Assessment Scale, *PSAS* Patient Scar Assessment Scale, *OSAS* Observer Scar Assessment Scale, *HWES* Hollander Wound Evaluating Score

^a^Indicates statistical significance

**Table 5:** Comparison of three groups in terms of patient satisfaction, LOS and Costs

**Characteristics**	**Group 1**	**Group 2**	**Group 3**	***P*-value**
	**(2-octyl)**	**(n-butyl-2)**	**(non-adhesive)**	
**Satisfaction, Likert scale**^a^				
**2 W**	4.41 ± 0.62	4.11 ± 0.80	3.61 ± 1.27	0.034^a^
**6 W**	4.31 ± 0.78	4.31 ± 0.68	4.12 ± 0.65	0.243
**3 M**	4.13 ± 1.18	3.60 ± 1.27	4.09 ± 0.78	0.073
**LOS**^b^ **(days)**	3.0(1)	4.0(1)	3.5(1)	0.013^c^
**Average cost in hospital** ^a^** (RMB)**	44.93 ± 33.97	126.50 ± 43.76	145.90 ± 67.70	< 0.001^c^
**Average cost of discharge** ^a^ **(RMB)**	0	619.43 ± 96.65	1902.50 ± 235.71	< 0.001^c^
**Total average cost** ^a^ **(RMB)**	1646.59 ± 33.97	1140.93 ± 102.55	2048.41 ± 228.49	< 0.001^c^

*IQR* interquartile range, *LOS* Length of stay

^a^Data are presented as means ± standard deviations

^b^Data are presented as median (IQR)

^c^Indicates statistical significance (statistically significant)

## Discussion

Periprosthetic joint infection (PJI) is a catastrophic complication that causes failure in some patients undergoing TKA and increases with the number of TKA. Persistent wound discharge after TKA is an important complication, which may potentially result in adverse consequences of PJI [2, 4, 18]. Protracted wound discharge and other wound complications increase the risk of PJI by 35 times [18, 35]. A study has shown that each additional day of wound discharge after TKA might increase the risk of wound infection by 29% [36]. Wound closure can affect the clinical outcomes after TKA, including the occurrence of wound complications [1–3]. The new skin adhesive octyl-2-cyanoacrylate has a sticky polyester mesh that is more resilient than previous skin adhesives or subcuticular sutures alone and can promote wound closure. The flexible mesh distributes tension evenly across the width of the mesh, rather than at a single anchor point, to ensure that the wound edges are better approximated and form a watertight barrier throughout the wound healing process, potentially preventing wound complications, including wound discharges [11, 12, 16]. This feature may be conducive to the healing of knee wounds since knee joints need extensive postoperative motion. Additionally, with octyl-2-cyanoacrylate, a dressing change is not required, allowing for painless glue removal. This may lower the risk of poor wound healing caused by friction between wound dressings and skin [11, 15–17, 19].

We quantitatively measured the wound discharge using photography software and found that using 2-octyl could effectively reduce the discharge area of the wound at 1, 3, and 5 days and the incidence of PWD after TKA, compared with non-adhesive group. The discharge area was smaller in n-butyl-2 group than in the non-adhesive group only at 1 day. This may be related to the increase in patient activity, such as knee flexion, at 3 and 5 days. More resilient characteristics enable 2-octyl to resist the high tension of the knee joint wound during exercise, thereby 2-octyl could reduce wound discharge area most effectively. No other complications developed in 2-octyl group. Allergic contact dermatitis (ACD), a wound-specific complication, caused by the use of skin adhesives has raised concerns, with reports showing that ACD occurred in about 0.5% of cases when skin adhesives containing 2-octyl-cyanoacrylate were used after orthopedic surgery [28, 37–40]. Nonetheless, in this study, we did not find ACD in 2-octyl group, which might be ascribed to the ease with which the adhesive was removed. 2-octyl can be completely removed about 2 weeks after operation. Another reason is that the sample size is not large enough, the former reduced the time of exposure to possible allergens [28]. However, one patient in the n-butyl-2 group developed ACD six weeks after operation, causing wound itching and dehiscence, resulting in poor wound appearance. We believe, in this case, ACD was caused by n-butyl-2 that lingered on the skin for a long time after operation. Once ACD occurs, it is difficult to remove, and scratching due to itching increases the risk of laceration and other complications. Therefore, great effort should be made to avoid ACD whenever possible. In the non-adhesive group, there was a case of PJI and huge blisters on the skin surface. The former may be caused by hematogenous infection due to decreased immunity of upper respiratory tract infection, but the presence of a small incision with pus on the wound surface suggested that PJI caused by local invasion of bacteria from the unprotected incision could not be ruled out. The latter was caused by the friction between the dressing and the skin when the incision was subjected to frequent dressing changes. Our results showed that the use of 2-octyl for TKA wound closure was pretty safe, and this finding was consistent with previous results [11, 19, 41]. It is worth pointing out that, in a randomized controlled trial of TKA incision closure that compared 2-octyl and subcuticular suture alone, the authors did not assess wound discharge complications and they found 4 cases of wound dehiscence (8%) in 50 patients who used 2-octyl and 3 of them required re-suturing, indicating that dehiscence was severe, against only one case of wound dehiscence in subcuticular suture group [11]. Although they believed that 2-octyl was equally safe, the wound complications are rare so this clinical result needs further optimization. The cause of the wound dehiscence might be attributed to the simple use of 2-octyl under higher knee tension without subcuticular sutures, leading to higher wound tension. In our study, 2-octyl was still used for subcuticular sutures and no dehiscence occurred.

Our study found that 2-octyl showed better cosmetic results at 6 weeks and 3 months. Wound assessment and repair at 3 months after injury were shown to be good measures of long-term cosmetic outcomes, and results at 3 months correlated well with those at 12 months [42]. So better long-term cosmetic outcomes were attained by using 2-octyl for TKA wound closure, which was consistent with previous results obtained in abdominal surgery [16]. Kavin et al. also showed that 2-octyl provided better wound cosmetology in TKA than staples [19]. Nevertheless, another study exhibited that wound cosmetic outcomes of only using 2-octyl were comparable to those of subcuticular sutures [11]. This difference might be explained by the additional tension caused by intensive knee rehabilitation after TKA, which was counteracted by subcuticular suture in Kavin et al.’s and our study, and the flexible multi-stick mesh was used to further reduce and evenly distribute the wound surface tension. The wound edges were therefore approximated to promote wound healing, ultimately meeting the need for rehabilitation while reducing scar formation [11]. Previous studies have demonstrated that using 2-octyl might achieve higher patient satisfaction compared to staple closure in TKA [19]; Our research showed that patient satisfaction was higher in 2-octyl group at 2 weeks compared to its non-adhesive counterpart.

Despite the added costs related to the use of skin adhesive, other costs were less compared to the non-adhesive group. Patients using the adhesive did not need dressing change after using 2-octyl, so the registration fee, dressing change fee, and transportation expenses caused by the wound care were saved. Compared with non-adhesive and n-butyl-2, the use of 2-octyl could reduce the average cost of wound care in hospitals by 100.97 and 81.57 RMB, respectively. Our results showed that the total cost of wound care was lower in the 2-octyl group than in the non-adhesive group. Although total cost was higher in the 2-octyl group than in the n-butyl-2 group, it did not include the additional transportation costs caused by the management of incision complications and remote re-examination. In addition, patients in 2-octyl group still had to change dressing within 5 days after surgery to evaluate wound discharge in this study, which increased some unnecessary costs. In the other groups, patients needed dressing change after discharge. This also caused great inconvenience to patients with mobility problems after TKA. Patients in 2-octyl group did not have to come back to the hospital for dressing changes, which saved patients' and doctors' time while avoiding the pain caused by dressing changes. Finally, although the use of skin adhesive resulted in about an additional 1 min of the operation time, it did not change the operation volume of the operating room in 1 day. It also did not generate additional operation costs in this country, because the cost of surgery has nothing to do with operation time in China. On the contrary, LOS was shorter in the patients using 2-octyl, compared to n-butyl-2, although we did not calculate this part of the cost due to the length of stay, the use of 2-octyl may shorten the length of stay, which will further reduce costs. Therefore, with TKA, the use of 2-octyl is convenient and cost-effective. In addition, the skin at the knee joint is often exposed, and as patients undergoing TKA become increasingly younger, the cosmetic effect of the wound is also a very important consideration for patients. Given the aforementioned factors, 2-octyl is a better choice in terms of cost-effectiveness.

In this study, strict inclusion and exclusion criteria were applied to minimize confounding factors. Malnutrition (serum albumin < 35 g/L) negatively affected wound healing, and these patients were more susceptible to deep infections [2, 43]. Although multiple comparisons showed that albumin on the first day was lower in 2-octyl group than in non-adhesive group (*P* = 0.008) in our study, the average albumin level was higher than 35 g at all times in all three groups. This finding further confirmed that 2-octyl could yield more favorable results in terms of lowered postoperative wound complication rate and improved wound healing.

This study has several limitations. First, although post-hoc power analysis showed our sample size sufficed to answer our first question (incidence of discharge), it may not be adequate to reveal significant differences in certain secondary endpoints, such as other complications. Second, we did not inform the follow-up doctors and patients of the wound closure method, but we could not guarantee that the medical staff involved in the follow-up were fully blinded since the wound with adhesive residues or early postoperative wound appearance looked different. Third, our study included patients receiving unilateral TKA who, unlike patients undergoing bilateral TKA, could not be self-controlled. However, our study endeavored to reduce a lot of confounding factors, and, considering that patients undergoing bilateral TKA were generally healthier and walked more slowly after surgery, these also impact wound healing. While our research results are more applicable to patients receiving unilateral TKA and we also compared LOS, which cannot be assessed in bilateral TKA. Fourth, although we have developed a unified postoperative wound management and rehabilitation program, it was difficult for a multicenter study to fully control all processes, which may also affect the results of the study. The multi-center nature of the study made our results more generalizable.

## Conclusion

Skin closure in TKA using 2-octyl adhesive material showed superiority to no skin adhesive or n-butyl-2. It reduced wound discharge and improved cosmetic outcomes, without increasing wound complications. In addition, the use of 2-octyl could achieve excellent patient satisfaction and lowered costs compared to no skin adhesive. Our study demonstrated that the use of 2-octyl was a safe and effective as a wound closure technique in patients undergoing TKA.

## Data Availability

The datasets generated and/or analyzed during the current study are not publicly available due to proprietary information.

## Abbreviations

2-octyl: 2-Octyl cyanoacrylate skin adhesive with flexible self-adhesive polyether mesh (DERMABOND PRINEO Ethicon, Inc)
n-butyl-2: N-butyl-2-cyanoacrylate skin adhesive (HISTOACRYL® B.Braun, Melsungen, German)
TKA: Total Knee Arthroplasty
LOS: Postoperative length of hospital stay
POSAS: Patient-Observer Scar Assessment Score
OSAS: Observer Scar Assessment Scale
PSAS: Patient scar assessment score
HWES: Hollander wound evaluating score
VSS: Vancouver scar scale
BMI: Body Mass Index
ACD: Allergic contact dermatitis
CCI: Charlson Comorbidity Index
PWD: Persistent Wound Drainage
PJI: Periprosthetic Joint Infection
Hb: Hemoglobin
Alb: Albumin
CRP: C-reactive protein
ESR: Erythrocyte sedimentation rate
PLT: Platelets
